# Supplementary material for: Effective Intervention Features of a Doping Prevention Program for Athletes: A Systematic Review with Meta-Analysis
Source: Sports (Basel). 2025 Apr 7;13(4):108. doi: 10.3390/sports13040108 (PMC12031626; doi:10.3390/sports13040108)
Supplement: Supplementary file 1 [file sports-13-00108-s001.zip › Table S1. Intervention description for coaches sample.pdf]

Table S1. Intervention characteristics of the study included in the systematic review with coach participants

**Reference**	**Population**	**Method**	**Intervention**	**Results**
Ntoumanis et al. [29], United Kingdom (UK), Greece and Australia	Coaches IG n = 62 (24.2% ♀, 75.8% ♂).<br>Age (M ±SD): 39.76 ±14.60 y.<br>Coaches CG n = 68 (26.5% ♀, 73.5% ♂).<br>Age (M ±SD): 38.82 ±12.15 y.<br>Sport level:<br>Not specified.	Study design: Cluster randomized controlled trial design with parallel group, two--condition, superiority trial. Three measures: Baseline (pre-intervention), end of intervention (12 weeks), and follow-up (2-months post-intervention).<br>Measures<br>1. Doping moral disengagement.<br>2. Attitudes toward doping.<br>3. Antidoping knowledge.<br>4. Efficacy to discuss doping issues.<br>5. Efficacy to create antidoping culture.<br>6. Encourage athletes to prevent inadvertent doping.<br>7. Perceived effectiveness of need support.<br>8. Perceived effectiveness of need thwarting.	Name of the intervention (IG): Motivational enrichment antidoping education.<br>Domain: Cognitive and affective.<br>Duration: Twelve 60 minutes sessions (one per week) for IG.<br>Characteristics: Standard antidoping education enriching with motivational content about the supportive communication style for coaches.<br>1. Standard antidoping education.<br>2. Introduction to the need supportive communication.<br>3. How to apply the need supportive communication to discuss doping-related issues with their athletes.<br>Coaches’ role: Active (IG).<br>Name of the intervention (CG): Standard antidoping education.<br>Domain: Cognitive.<br>Characteristics: ; A 60-minute single-session using the WADA-NADO Standard antidoping education program.<br>Coaches’ role: Passive.	Doping moral disengagement (M ±SD)<br>↔ IG (T1 = 1.40 ± 0.66; T2 = 1.27 ± 0.70) *vs*. CG (T1 = 1.34 ± 0.49; T2 = 1.33 ± 0.64).<br>↔ IG (T2 = 1.27 ± 0.70; T3 = 1.20 ± 0.30) *vs*. CG (T2 = 1.33 ± 0.64; T3 = 1.24 ± 0.42).<br>Attitudes toward doping (M ±SD)<br>↓* IG (T1 = 1.29 ± 0.43; T2 = 1.18 ± 0.42) *vs*. CG (T1 = 1.38 ± 0.68 ; T2 = 1.30 ± 0.49).<br>↔ IG (T2 = 1.18 ± 0.42; T3 = 1.18 ± 0.32) *vs*. CG (T2 = 1.30 ± 0.49; T3 = 1.33 ± 0.45).<br>Efficacy to discuss doping issues (M ±SD)<br>↔ IG (T1 = 75.13 ± 19.36; T2 = 85.17 ± 17.11) *vs*. CG (T1 = 78.99 ± 15.30; T2 = 84.73 ± 11.32).<br>↔ IG (T2 = 85.17 ± 17.11; T3 = 87.81 ± 11.99) *vs*. CG (T2 = 84.73 ± 11.32; T3 = 87.84 ± 10.93).<br>Efficacy to create antidoping culture (M ±SD)<br>↑* IG (T1 = 81.63 ± 18.03; T2 = 90.17 ± 11.28) *vs*. CG (T1 = 83.90 ± 15.77 ; T2 = 86.68 ± 11.13).<br>↔ IG (T2 = 90.17 ± 11.28; T3 = 91.02 ± 8.44) *vs*. CG (T2 = 86.68 ± 11.13; T3 = 89.23 ± 10.51).<br>Encourage athletes to prevent inadvertent doping (M ±SD)<br>↓* IG (T1 = 0.79 ± 1.49; T2 = 2.94 ± 2.18) *vs*. CG (T1 = 1.24 ± 1.89 ; T2 = 2.47 ± 2.09).

↔ IG (T2 = 2.94 ± 2.18; T3 = 2.40 ± 2.01) *vs*. CG (T2 = 2.47 ± 2.09; T3 = 2.96 ± 2.13).
Antidoping knowledge (M ±SD)
↔ IG (T1 = 3.59 ± 1.43; T2 = 4.68 ± 1.25) *vs*. CG (T1 = 3.74 ± 1.46; T2 = 5.02 ± 0.83).
↔ IG (T2 = 4.68 ± 1.25; T3 = 4.96 ± 0.98) *vs*. CG (T2 = 5.02 ± 0.83; T3 = 5.15 ± 0.82).
Perceived effectiveness of need support (M ±SD)
↑* IG (T1 = 5.79 ± 0.90; T2 = 6.22 ± 0.76) *vs*. CG (T1 = 5.81 ± 1.08 ; T2 = 5.86 ± 0.78).
↔ IG (T2 = 6.22 ± 0.76; T3 = 6.21 ± 1.04) *vs*. CG (T2 = 5.86 ± 0.78; T3 = 6.05 ± 0.90).
Perceived effectiveness of need thwarting (M ±SD)
↓* IG (T1 = 2.39 ± 0.80; T2 = 1.88 ± 1.17) *vs*. CG (T1 = 2.54 ± 1.11 ; T2 = 2.36 ± 1.12).
↑* IG (T2 = 1.88 ± 1.17; T3 = 2.02 ± 1.30) *vs*. CG (T2 = 2.36 ± 1.12; T3 = 1.98 ± 0.99).

Note. ♀: women; ♂: men; y: Years; IG: intervention group; CG: control group; M: Mean; SD: Standard Deviation; ↓: decrease; ↑: increase; ↔: not significant change; *: statistically significant change ($p < .05$); T1: Evaluation before intervention program (pre); T2: Evaluation after intervention program (post); T3: Follow-up number 1 after post-intervention evaluation.
