# Supplementary material for: Effective Intervention Features of a Doping Prevention Program for Athletes: A Systematic Review with Meta-Analysis
Source: Sports (Basel). 2025 Apr 7;13(4):108. doi: 10.3390/sports13040108 (PMC12031626; doi:10.3390/sports13040108)
Supplement: Supplementary file 1 [file sports-13-00108-s001.zip › Table S2. Codes of intervention groups per variable for Doping intention in Meta-analysis.pdf]

Table S2. Intervention group codes by variable in the meta-analysis of doping intentions.

Author	Variable	Group	Symbol
Ntoumanis et al. (2020)[43]	Doping willingness	Motivational enrichement anti-doping education	a
Ntoumanis et al. (2020)[43]	Doping willingness	Standard anti-doping education	b
Ntoumanis et al. (2020)[43]	Attitudes toward doping	Motivational enrichement anti-doping education	c
Ntoumanis et al. (2020)[43]	Attitudes toward doping	Standard anti-doping education	d
Manges et al. (2022)[44]	Doping susceptibility	Based-values intervention	a
Manges et al. (2022)[44]	Doping susceptibility	Information-based intervention	b
Manges et al. (2022)[44]	Doping intention	Based-values intervention	c
Manges et al. (2022)[44]	Doping intention	Information-based intervention	d
Nicholls et al. (2020)[45]	Doping attitudes	Presentation	a
Nicholls et al. (2020))[45]	Doping attitudes	Online I Play Clean	b
Nicholls et al. (2020))[45]	Doping attitudes	Presentation + Online I Play Clean	c
Nicholls et al. (2020))[45]	Doping susceptibility	Presentation	d
Nicholls et al. (2020))[45]	Doping susceptibility	Online I Play Clean	f
Nicholls et al. (2020))[45]	Doping susceptibility	Presentation + Online I Play Clean	g
Kavussanu et al. (2021) (UK)[23]	Doping likelihood	Moral Intervention	a
Kavussanu et al. (2021) (UK)[23]	Doping likelihood	Educational intervention	b
Kavussanu et al. (2021) (Gr)[23]	Doping likelihood	Moral Intervention	c
Kavussanu et al. (2021) (Gr)[23]	Doping likelihood	Educational intervention	d
Kavussanu et al. (2022)[16]	Doping likelihood	Psychological intervention	a
Kavussanu et al. (2022)[16]	Doping likelihood	Educational intervention	b
Galli et al. (2022)[46]	Doping likelihood	Serious game (video game)	
Deng et al. (2022)[48]	Attitudes toward doping	Athlete Learning Program about Health and Anti-Doping (ALPHA)	a
Deng et al. (2022)[48]	Doping likelihood (benefical situation)	Athlete Learning Program about Health and Anti-Doping (ALPHA)	b
Deng et al. (2022)[48]	Doping likelihood (cost situation)	Athlete Learning Program about Health and Anti-Doping (ALPHA)	c
Hurtst et al. (2023)[47]	Doping susceptibility	UK Anti-Doping Clean Sport education program	a
Hurtst et al. (2023)[47]	Dietary supplementation intention	UK Anti-Doping Clean Sport education program	b
Thomas et al. (2023)[39]	Attitudes toward doping	Educational Flyer	
