# Supplementary material for: Effective Intervention Features of a Doping Prevention Program for Athletes: A Systematic Review with Meta-Analysis
Source: Sports (Basel). 2025 Apr 7;13(4):108. doi: 10.3390/sports13040108 (PMC12031626; doi:10.3390/sports13040108)
Supplement: Supplementary file 1 [file sports-13-00108-s001.zip › Table S3. Codes for Intervention Groups by Variable in the Anti-doping behaviors Meta-analysis..pdf]

Table S3. Intervention group codes by variable in the meta-analysis of Anti-Doping Behaviors.

Author	Variable	Group	Symbol
Ntoumanis et al. (2020)[43]	Efficacy to resist doping	Motivational enrichement anti-doping education	a
Ntoumanis et al. (2020)[43]	Efficacy to resist doping	Standard anti-doping education	b
Ntoumanis et al. (2020)[43]	Behaviors against involuntary doping	Motivational enrichement anti-doping education	c
Ntoumanis et al. (2020)[43]	Behaviors against involuntary doping	Standard anti-doping education	d
Ntoumanis et al. (2020)[43]	Antidoping knowledge	Motivational enrichement anti-doping education	e
Ntoumanis et al. (2020)[43]	Antidoping knowledge	Standard anti-doping education	f
Kavussanu et al. (2022)[16]	Self-regulatory efficacy	Psychological intervention	a
Kavussanu et al. (2022)[16]	Self-regulatory efficacy	Educational intervention	b
Galli et al. (2022)[46]	Self-regulatory efficacy	Serious game (video game)	a
Galli et al. (2022)[46]	Anti-doping knowledge	Serious game (video game)	b
Deng et al. (2022)[48]	Anti-doping knowledge	Athlete Learning Program about Health and Anti-Doping (ALPHA)	
Hurtst et al. (2023)[47]	Anti-doping knowledge	UK Anti-Doping Clean Sport education program	a
Hurtst et al. (2023)[47]	Anti-doping practice	UK Anti-Doping Clean Sport education program	b
Hurtst et al. (2023)[47]	Whistleblowing	UK Anti-Doping Clean Sport education program	c
Da Silva et al. (2019)[40]	Positive knowledge	Hearth in game	a
Da Silva et al. (2019)[40]	Negative knowledge	Hearth in game	b
