# Supplementary material for: Effective Intervention Features of a Doping Prevention Program for Athletes: A Systematic Review with Meta-Analysis
Source: Sports (Basel). 2025 Apr 7;13(4):108. doi: 10.3390/sports13040108 (PMC12031626; doi:10.3390/sports13040108)
Supplement: Supplementary file 1 [file sports-13-00108-s001.zip › Table S4. Intervention group codes by variable in the meta-analysis of Anti-doping moral behavior..pdf]

Table S4. Intervention group codes by variable in the meta-analysis of Anti-doping moral behavior.

Author	Variable	Group	Symbol
Ntoumanis et al. (2020)[43]	Moral disengagement (-)	Motivational enrichement anti-doping education	a
Ntoumanis et al. (2020)[43]	Moral disengagement (-)	Standard anti-doping education	b
Manges et al. (2022)[44]	Moral disengagement (-)	Based-values intervention	a
Manges et al. (2022)[44]	Moral disengagement (-)	Information-based intervention	b
Manges et al. (2022)[44]	Anticipated guilt (+)	Based-values intervention	c
Manges et al. (2022)[44]	Anticipated guilt (+)	Information-based intervention	d
Manges et al. (2022)[44]	Empathy (+)	Based-values intervention	e
Manges et al. (2022)[44]	Empathy (+)	Information-based intervention	f
Manges et al. (2022)[44]	Collective moral norms (+)	Based-values intervention	g
Manges et al. (2022)[44]	Collective moral norms (+)	Information-based intervention	h
Kavussanu et al. (2021) (UK)[23]	Moral Identity (+)	Moral Intervention	a
Kavussanu et al. (2021) (UK)[23]	Moral Identity (+)	Educational intervention	b
Kavussanu et al. (2021) (Gr)[23]	Moral Identity (+)	Moral Intervention	c
Kavussanu et al. (2021) (Gr)[23]	Moral Identity (+)	Educational intervention	d
Kavussanu et al. (2021) (UK)[23]	Moral disengagement (-)	Moral Intervention	e
Kavussanu et al. (2021) (UK)[23]	Moral disengagement (-)	Educational intervention	f
Kavussanu et al. (2021) (Gr)[23]	Moral disengagement (-)	Moral Intervention	g
Kavussanu et al. (2021) (Gr)[23]	Moral disengagement (-)	Educational intervention	h
Kavussanu et al. (2021) (UK)[23]	Moral atmosphere (+)	Moral Intervention	i
Kavussanu et al. (2021) (UK)[23]	Moral atmosphere (+)	Educational intervention	j
Kavussanu et al. (2021) (Gr)[23]	Moral atmosphere (+)	Moral Intervention	k
Kavussanu et al. (2021) (Gr)[23]	Moral atmosphere (+)	Educational intervention	l
Kavussanu et al. (2021) (UK)[23]	Anticipated guilt (+)	Moral Intervention	m
Kavussanu et al. (2021) (UK)[23]	Anticipated guilt (+)	Educational intervention	n
Kavussanu et al. (2021) (Gr)[23]	Anticipated guilt (+)	Moral Intervention	o
Kavussanu et al. (2021) (Gr)[23]	Anticipated guilt (+)	Educational intervention	p
Kavussanu et al. (2022)[16]	Moral disengagement (-)	Psychological intervention	a
Kavussanu et al. (2022)[16]	Moral disengagement (-)	Educational intervention	b
Kavussanu et al. (2022)[16]	Anticipated guilt (+)	Psychological intervention	c
Kavussanu et al. (2022)[16]	Anticipated guilt (+)	Educational intervention	d
Galli et al. (2022)[46]	Moral disengagement (-)	Serious game (video game)	
Hurtst et al. (2023)[47]	Spirit of the sport values (+)	UK Anti-Doping Clean Sport education program	a
Hurtst et al. (2023)[47]	Moral values (+)	UK Anti-Doping Clean Sport education program	b

Note. (+) = Positive anti-doping moral behavior variable; (-) = Negative anti-doping moral behavior variable.
