# Supplementary figures and images for: Effective Intervention Features of a Doping Prevention Program for Athletes: A Systematic Review with Meta-Analysis
Source: Sports (Basel). 2025 Apr 7;13(4):108. doi: 10.3390/sports13040108 (PMC12031626; doi:10.3390/sports13040108)

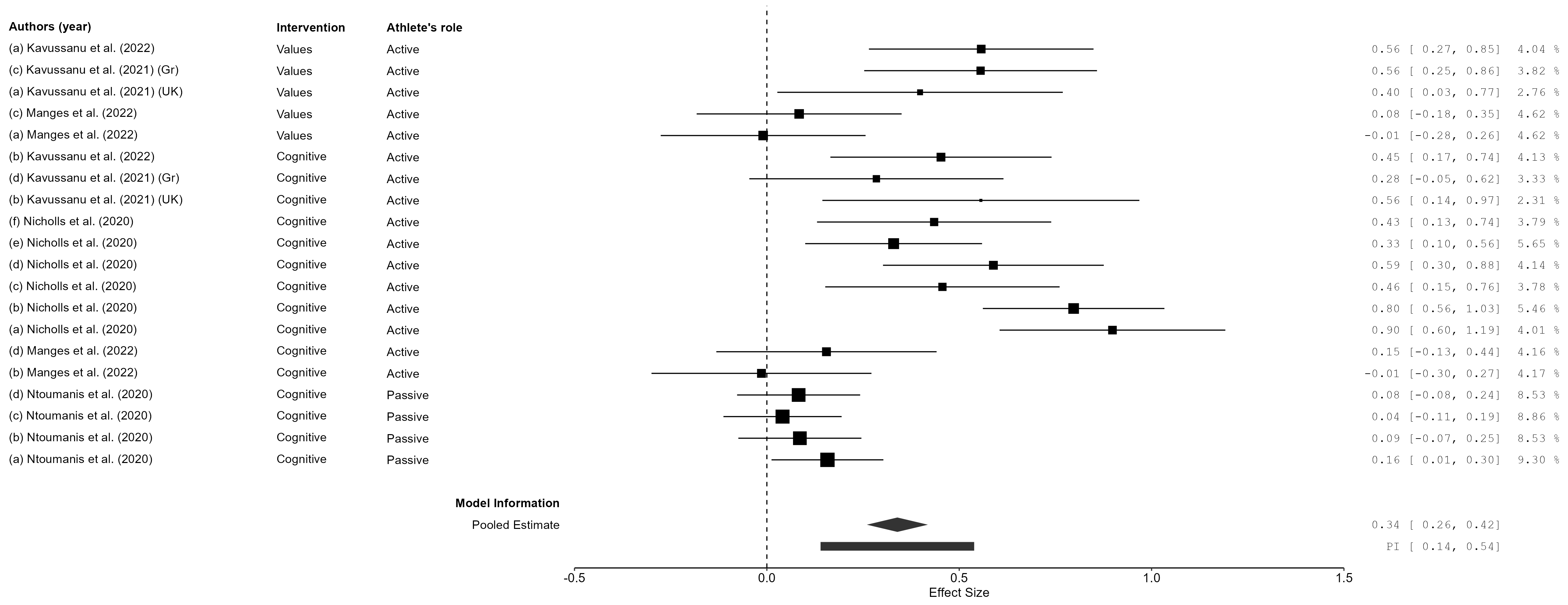

Supplement: Supplementary file 1 [file sports-13-00108-s001.zip › Figure S1. Doping intention Pre-Follow up.png]

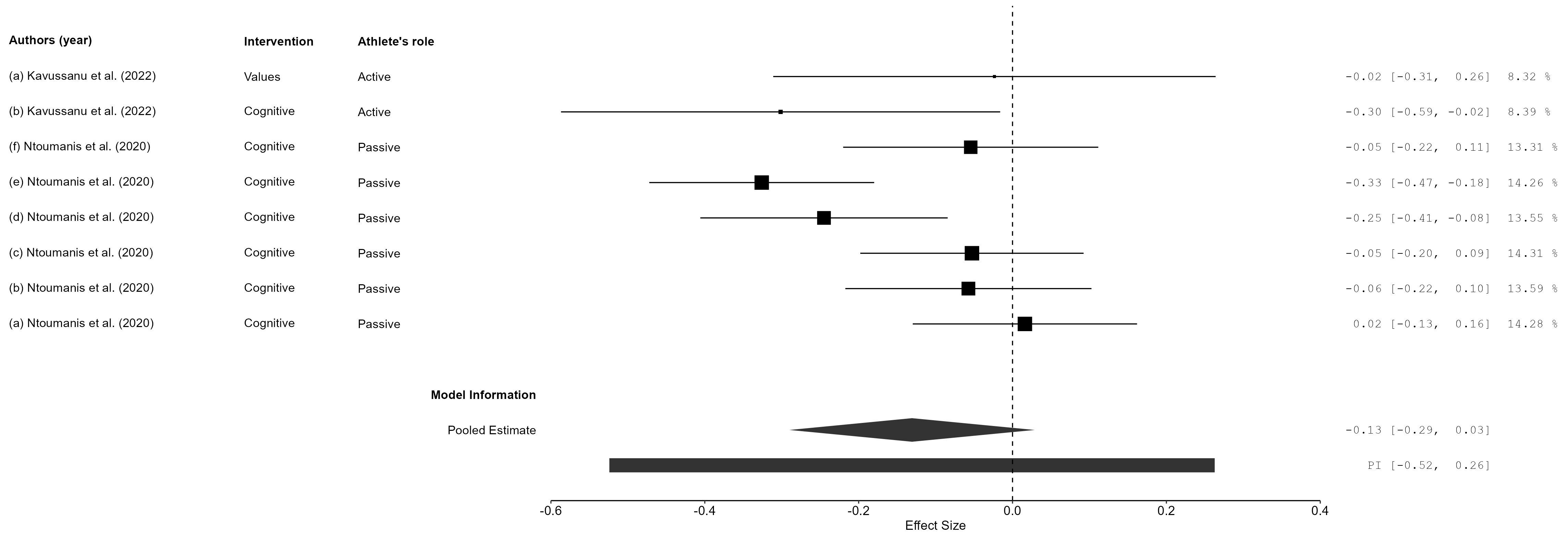

Supplement: Supplementary file 1 [file sports-13-00108-s001.zip › Figure S2. Anti-doping behavior Pre-Follow up.png]

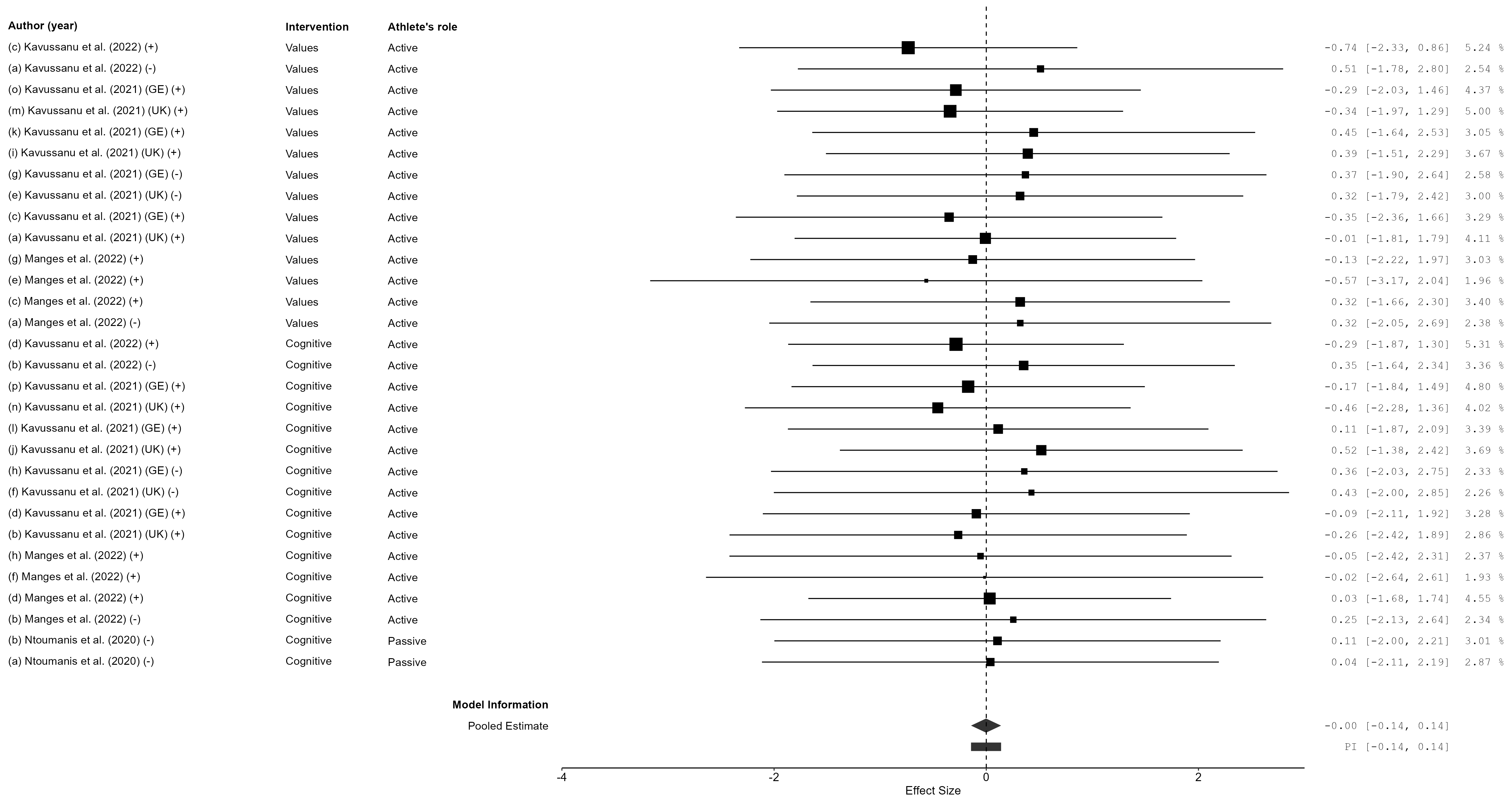

Supplement: Supplementary file 1 [file sports-13-00108-s001.zip › Figure S3. Anti-Doping Moral Behavior Pre - Follow up.png]
